# Septal Myectomy in Patients with Hypertrophic Cardiomyopathy and Nonclassical Anderson–Fabry Disease

**DOI:** 10.3390/jcdd11090293

**Published:** 2024-09-20

**Authors:** Alexandr Gurschenkov, Sofiya Andreeva, Vadim Zaitsev, Pavel Khazov, Gleb Ischmukhametov, Alexandra Kozyreva, Polina Sokolnikova, Olga Moiseeva, Anna Kostareva, Mikhail Gordeev

**Affiliations:** 1 Institute of Cardiovascular Science, Almazov National Medical Research Centre, 197341 Saint Petersburg, Russia; 2 World-Class Research Centre for Personalized Medicine, Almazov National Medical Research Centre, 197341 Saint Petersburg, Russia; 3 Department of Woman and Children’s Health, Karolinska Institutet (KI), 171 77 Stockholm, Sweden

**Keywords:** hypertrophic cardiomyopathy, obstructive, myectomy, Anderson–Fabry disease, *GLA*

## Abstract

Anderson–Fabry disease (AFD) results from decreased enzyme activity of lysosomal enzymes and intralysosomal storage of nonhydrolyzed forms. Cardiovascular complications, mainly in the form of HCM, contribute substantially to AFD patient mortality. Here, we report three new cases of obstructive HCM (HOCM) in nonclassical presentations of AFD and isolated cardiac involvement. In all three cases, the diagnosis of AFD was made postoperatively by routine genetic and morphological testing. Together with previously published cases, this report illustrates the potential safety and beneficial effect of septal surgical myectomy in patients with AFD-HOCM, as well as underlines the need for more thorough screening for clinical signs of AFD-associated cardiomyopathy and *GLA* variants among patients with HOCM.

## 1. Introduction

The *GLA* gene encodes for the α-galactosidase enzyme, and its over 850 pathogenic and likely pathogenic variants are all associated with the only clinical phenotype—Anderson–Fabry disease (AFD). This disease results from decreased enzyme activity and intralysosomal storage of a nonhydrolyzed substrate—globotriaosylceramide [1,2]. The cells and tissues that suffer the most are the heart, kidney, and nervous system, as well as eyes and ears due to endothelial damage and intracellular storage of undegraded substrates [3]. The classical form of the disease progresses permanently, leading to substantially decreased quality of life and life expectancy [4]. Cardiovascular complications contribute substantially to AFD patient mortality, and half the patients develop left ventricular hypertrophy within 15 years of follow-up accompanied by all associated risk factors [5,6]. Apart from the classical form with typical presentation and systemic organ damage, there have been nonclassical and non-penetrating forms with late manifestation and isolated organ involvement described—mainly the cardiovascular system or kidneys. These cases constitute a substantial diagnostic challenge, often leaving the patient without correct clinical or genetically proved diagnoses, enzyme replacement therapy, or proper risk stratification. Thus, despite the fact that hypertrophic cardiomyopathy is a well-known cardiovascular presentation of Fabry disease in the form of subaortic, midventricular, or apical hypertrophic remodeling, there are few reports on the obstructive form of AFD-HCM and surgical septal myectomy (SSM) [7,8,9,10,11,12,13,14]. In most reported cases, the patients were admitted to surgical treatment with an already established diagnosis of AFD, and only in a few cases were patients treated as having classical HCM, with the precise diagnosis becoming obvious only after cardiac surgery. Here, we report three new cases of obstructive HCM due to nonclassical presentation of AFD and isolated cardiac involvement. In all three cases, the diagnosis of AFD was made postoperatively by routine genetic and morphological testing. In addition, we summarize all previously published cases of SSM in patients with AFD, providing a summary on safety and benign prognoses following such operations in patients with AFD. In addition to previously reported cases, our series underlines the safety and effectiveness of SSM in obstructive form of AFD.

## 2. Case Reports

Three patients were enrolled at the Almazov National Medical Research Centre during 2016–2022 to have SSM performed due to an obstructive form of HCM without a known previous diagnosis of AFD. The main clinical characteristics of the patients are summarized in Table 1. In all patients, standard clinical examination including echocardiography and Holter electrocardiogram (ECG) monitoring were performed prior to cardiac surgery (Table 2). MRI images were obtained using ultrahigh-field tomography with a Magnetom Trio A Tim 3.0 Tл (Siemens, Munich, Germany) with 8 mm slices using Gd-DO3A 0.2 mL/kg contrast. Of note, two of the patients underwent non-ST MI prior to operation, and the third patient had classical angina without any intracoronary obstruction based on angiography. In one patient, a pacemaker was implanted due to second-degree AV block (type 2). None of the patients revealed ventricular tachycardia, and all three presented with premature grade III–IV ventricular contractions. All patients revealed various degrees of myocardial fibrosis on magnetic resonance imaging (MRI), either in both ventricles (Patient 1), in the interventricular septum (IVS) and left ventricle (LV), (Patient 2) or solely in the LV (Patient 3). Only Patient 1 had increased right ventricular thickness. All surgical procedures were performed with cardiac arrest under retrograde Calafiore blood cardioplegia with modification [15]. In one case (Patient 1), the superior vena cava was dissected in order to verticalize the interventricular septum due to poor visualization (Table 3). In Patient 3, mitral second-order chordae resection of the anterior mitral valve leaflet was performed [16]. None of the three patients had postsurgical complications and were discharged on day 14–16 with remarkable clinical and subjective improvement (chronic heart failure (CHF) class I–II and no signs of angina). Morphological examination confirmed extensive fibrosis and disarray.

**Figure 1:**
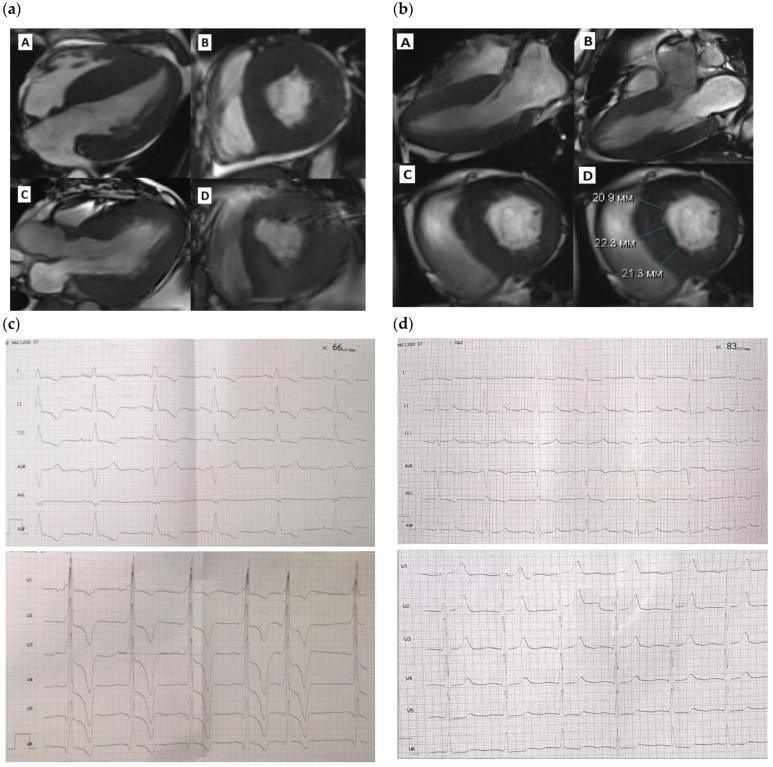
Cardiac MRI before and after SSM. (**a**)—Patient 1, A—diastolic view before SSM, long left ventricular axis, B—diastolic view before SSM, short left ventricular axis, C—diastolic view after SSM, long left ventricular axis, D—diastolic view after SSM, short left ventricular axis; (**b**)—Patient 3, A,B—diastolic view before SSM, long left ventricular axis, C,D—diastolic view before SSM, short left ventricular axis; (**c**)—Patient 1, ECG; (**d**)—Patient 3, ECG.

All patients were alive 12 and 18 months postoperatively and remained on NYHA class II (Patients 1 and 2) and class I (Patient 3). The data on pre- and post-intervention NT-proBNP levels were available only for Patient 1 and demonstrated a marked postoperative improvement (from 11,568.00 pg/mL to 4326.00 pg/mL on day 7 after surgery). Of note, in all three patients, CHF symptoms along with elevated NT-proBNP level persisted at one year after surgery despite a marked reduction in left ventricular outflow tract (LVOT) gradient. In addition, none of the patients demonstrated abnormal PVC numbers or had indications for implantable cardioverter–defibrillator (ICD) implantation in spite of a severe LVOT gradient prior to surgery (Figure 1).

## 3. Discussion

In spite of the well-known fact that AFD often manifests with a cardiac phenotype in a form of HCM, the diagnostic workup in cases with atypical AFD with only cardiovascular symptoms remains a challenge. Importantly, since the use of an HCM risk calculator is not validated for patients with storage diseases, no straightforward clinical guidelines for ICD implantation can be used in patients with identified *GLA* mutations. The same is valid for other treatment strategies of HCM in cases of metabolic and storage disorders. This group of patients drops off the current guidelines and treatment algorithms, since these patients fully manifest neither classical signs of AFD phenotype nor HCM clinical cause. For this reason, a newly proposed staging for AFD-associated HCM was recently offered to better identify the treatment strategy, surgical risks, and patient prognosis [17]. Another staging system to define a prognosis of AFD patients independent of surgical intervention was proposed by Meucci and co-authors; however, the role of LVOT obstruction was not among the parameters analyzed [6]. Currently, AFD contributes to only a small proportion of HCM, approximately 0.4–1% [13,18,19,20]. However, with implementation of routine genetic testing in HCM diagnostics, the number of reported patients with nonclassical AFD among HCM patients is constantly increasing, including a group of surgically treated patients. Thus, among patients with HOCM, AFD is reported to contribute to 0.5–2% [12]. Several interventional and surgical options can be offered to patients with HOCM, including myectomy and septal alcohol ablation [21]. Myectomy also aims to reduce LVOT gradient, relieving exercise intolerance and improving HF symptoms in patients with LVOT obstruction. The last statement was confirmed in our case series, where all three patients showed improved NYHA class from III to II and I. Of note, in a growing number of cases, a definitive diagnosis of AFD is established only during surgical operation due to the surgeon’s attention to myocardial tissue structure, meaning that a number of patients with HOCM do not have any red flags of AFD prior to surgery [11]. As an option, an ECG-based calculator can be used to identify the MRI signs of AFD [22,23]. If used in Patient 1, this calculator might have helped identify the etiology of LVOTO early, before surgical intervention. However, storage disorders are often considered a nontarget group for myectomy, since no systematic data, review, or meta-analysis has been performed on the effectiveness of surgical treatment or the postoperative course in this group of patients.

Several case reports have been described for patients with AFD who underwent surgical myectomy. Together with the 3 patients presented in this study, 22 patients overall who underwent open surgery treatment due to HOCM and AFD have now been reported (Table 4). In almost half the cases, the diagnosis of AFD was established prior to surgery, and 9 out of 22 patients obtained enzyme replacement therapy.

Despite the well-documented effect of enzyme replacement therapy (ERT) on organ damage and disease progression [24], its protective effect on cardiomyopathy and relieving HF symptoms in patients with AFD is not obvious [25,26]. One of the explanations can be related to the possible immune and cell-stress-mediated mechanisms of cardiomyocyte injury and hypertrophy in AFD cardiomyopathy, which once induced can persist for long time in spite of the absence of initial metabolic alterations and effective ERT [25,27]. This notion can be supported by the fact that 9 out of 22 patients who had undergone myectomy had ERT and nevertheless reached the point of indication for surgical treatment due to progression of hypertrophy and obstruction. Data exist regarding the most beneficial effect of ERT or chaperone therapy with migalastat on cardiac function in patients with very early stage of cardiac involvement and the decreased effect of specific therapy on cardiac function in patients with advanced cardiomyopathy and hypertrophy [25,27,28,29]. Importantly, the molecular mechanisms of cardiac-only AFD can be slightly different from cases with full disease penetrance and classical presentations. This is probably associated with a defined genetic alteration and the functional effect of a variant of enzyme activity and function. Thus, the Ala143Thr variant described in this study has been widely debated as a possible cause of the full-penetrance phenotype of AFD and has been demonstrated to be associated with only late-onset cardiomyopathy with incomplete penetrance [30,31,32], similarly for the genetic variants that lead to the formation of cryptic splice sites and inclusion of additional exons [33,34,35]. These low-penetrance *GLA* variants and variants in noncoding regions that are not always covered by target gene panels must be considered in patients with HOCM as a possible cause of the AFD cardiac-only phenotype.

## 4. Conclusions

In conclusion, we described three new cases of successful SSM in patients with HOCM due to AFD. In all three patients, the genetic diagnosis was established only after surgery, since they did not have other classical symptoms of AFD. Together with previously published cases, this report illustrates the potential safety and beneficial effects of SSM in patients with AFD-HOCM, as well as underlines the need for more thorough screening for clinical signs of AFD-associated cardiomyopathy and *GLA* variants among patients with HOCM.

## Figures and Tables

**Table 1:** Clinical characteristics of HOCM patients before myectomy.

Clinical Parameter	Patient 1	Patient 2	Patient 3
Sex	male	female	female
Age at diagnosis of HOCM	51	69	58
Hight/Weight/BMI	174/66/21.8	162/90/34.3	182/96/29.0
Genetic variant, hg19	chrX:100653857T>C, c.717A>G, Ile239Met; NM_000169.3; rs1928192530	chrX:100656740C>T, c.427G>A, Ala143Thr NM_000169.2; rs104894845	chrX:100654790T>C c.640-856A>G; NM_000169.3
α-GAL A activity on leukocytes, nmol/mL/h	−	5.46	−
Plasma LysoGb3 level	−	0.43	0.78
NYHA at first evaluation	III	III	III
AF	+	−	−
Angina	+	−	−
History of MI	+ (non-ST)	+ (non-ST)	−
Coronary artery obstruction	−	−	−
Pacemaker	−	+	−
Kidney involvement	+	+	−
Angiokeratomas	−	−	−
Neurological symptoms	−	−	−

BMI—body mass index, LysoGb3—globotriaosylsphingosine, AF—atrial fibrillation, MI—myocardial infarction.

**Table 2:** Echocardiographic data of HOCM patients before myectomy.

Clinical Parameter	Case 1	Case 2	Case 3
IVS, mm	32	25	17
LVPW (d), mm	26	16	12
LVOT max gradient, mmHg	112	130	110
LA, mm	52	−	−
LV EF, %	74	78	73
RVW (d), mm	9	5	4
TAPSE	22	21	20
SAM +\−	+	+	+
MR	0	I–II	II–III
E/A	0.57	4.0	3.0
Type of diastolic dysfunction	1	3	3

IVS—interventricular septum, LVPW (d) left ventricular posterior wall (diastolic dimension), LVOT—left ventricular outflow tract obstruction, LA—left atrium, LV EF—left ventricular ejection fraction, RVW (d)—right ventricular wall (diastolic dimension), TAPSE—tricuspid annulus plane systolic motion, SAM—systolic anterior motion of mitral valve, MR—mitral regurgitation.

**Table 3:** Intraoperative characteristics of patients with HOCM during SSM.

Intraoperative Characteristics	Patient 1	Patient 2	Patient 3
Type of surgery	Extended myectomy	Extended myectomy	Extended myectomy + MV plastic
Time of circulatory arrest (min)	70	62	60
Time of aorta clip (min)	43	55	49
Excised myocardial mass (g)	6.37	8.1	3.41
IVS thickness at subaortic level (mm)	16	16	10
LV maximum thickness (mm)	31	16	15
Maximum LVOT gradient	30	12.9	11.9
LV EF % at day 7 postoperatively	71	69	63
MR	0	I	0

MV—mitral valve IVS—interventricular septum; LV—left ventricle; LVOT—left ventricular outflow tract; LV EF—left ventricular ejection fraction.

**Table 4:** Summary of cases with AFD with surgical septal myectomy.

	Diagnosis Established before Surgery	Age at Myectomy	Sex	Non-Cardiac Manifestations	ERT	LVOT Gradient	Post-Surgery Complications	Successful Discharge	Reference
1	+	53	m	+	+	100	−	+	Meghji [7]
2	−	37	f	−	−	75	−	+	
3	+	44	f	+	+	95	−	+	
4	+	41	f	+	−	174	−	+	
5	−	59	f	−	−	121	+ (stroke)	+	
6	−	72	f	−	−	67	−	+	
7	+	57	f	+	+	81	−	+	
8	+	56	f	+	+	152	−	+	Calcagnino [14]
9	+	n/a	f	+	+	56/58	−	+	
10	−	59	m	−	−	100	+ (LV disfunction, acute kidney injury)	+	Frustaci [12]
11	−	67	m	−	−	100	−	+	Cecchi [11]
12	−	56	m	−	−	70	−	+	
13	−	65	m	−	−	120	+ (VT)	+	
14	+	54	m	+	+	100	−	+	Kunkola [8]
15	+	44	f	−	+	95	−	+	
16	+	38	m	+	+	190	−	+	Raju [9]
17	+	55	n/a	−	−	85	+ (AV block)	+	Xiao [13]
18	+	49	n/a	+	−	88	−	+	
19	+	46	m	−	+	90	−	+	Blount [10]
20	−	51	m	+	−	112	−	+	Current study
21	−	69	f	+	−	130	−	+	
22	−	58	f	−	−	110	−	+	

AFD—Anderson–Fabry disease, ERT—enzyme replacement therapy, LVOT—left ventricular outflow tract, LV—left ventricle, VT—ventricular tachycardia, AV block—atrioventricular block.

## Data Availability

The datasets generated and analyzed for this study can be requested from the corresponding author.
